# Interconnected associations of occupational burnout, anxiety, and sleep quality in oilfield workers

**DOI:** 10.3389/fpubh.2025.1723075

**Published:** 2026-01-12

**Authors:** Ziwei Guo, Xuefeng Yu, Haobiao Liu, Qingsong Li, Licheng Yang, Xining Wang, Abebe Feyissa Amhare, Guoqiang Dong, Jing Han

**Affiliations:** 1 Xi'an Gem Flower Changqing Hospital, Xi'an, Shaanxi, China; 2 Department of Occupational and Environmental Health, School of Public Health, Health Science Center, Xi'an Jiaotong University, Xi'an, Shaanxi, China; 3 Department of Comprehensive Orthopedics, the Second Affiliated Hospital of Xi'an Jiaotong University, Xi'an, Shaanxi, China; 4 Institute for Hygiene of Ordnance Industry, Xi'an, Shaanxi, China

**Keywords:** anxiety, cross-sectional study, occupational burnout, oilfield workers, sleep quality

## Abstract

**Background:**

Occupational burnout and poor sleep quality are increasingly prevalent among oilfield workers, exposed to high-stress environments and irregular shifts. Emerging evidence indicates that anxiety may mediate the link between burnout and sleep disturbances, though empirical data in this group remain limited.

**Methods:**

A cross-sectional survey was conducted among 1,617 oilfield workers in Shaanxi Province, China. Sleep quality, anxiety, and occupational burnout were assessed using the Pittsburgh Sleep Quality Index, Symptom Checklist-90, and Maslach Burnout Inventory–General Survey, respectively. Logistic regression, correlation analysis, restricted cubic spline models, and bootstrapped mediation analyses were used to evaluate associations and mediation effects.

**Results:**

Among participants, 52.75% experienced occupational burnout. In the total population, occupational burnout were significantly associated with sleep quality (*OR* = 1.611, 95% CI: 1.291–2.010, *P* < 0.001). Sex-stratified analyses yielded consistent findings, with females showing stronger associations (*OR* = 1.919, 95% CI: 1.244–2.959) compared with males (*OR* = 1.492, 95% CI: 1.144–1.946). Restricted cubic spline analysis revealed a nonlinear dose–response relationship between occupational burnout and the odds of sleep quality (*P* for nonlinear = 0.002). Mediation analysis showed that anxiety is consistent with a significant indirect association between occupational burnout and sleep quality, accounting for 33.08% of the total association.

**Conclusions:**

Occupational burnout and anxiety are key correlates for sleep quality among oilfield workers, with anxiety serving as a significant potential mechanism. Early identification and targeted interventions addressing both occupational burnout and psychological distress are important considerations for improving sleep quality and alleviating occupational health in high-risk work environments.

## 1 Introduction

Occupational burnout and poor sleep quality are widely recognized as major public health challenges, with substantial consequences for physical and mental well-being across diverse occupations. Recent meta-analyses indicate that occupational burnout is common across various industries, affecting a considerable proportion of the workforce worldwide (1–3). Poor sleep quality, including insomnia and circadian rhythm disruption, further exacerbate psychological distress and health risks, forming a reciprocal relationship with occupational strain (4, 5).

Oilfield workers represent a unique high-risk occupational group warranting specific attention. Unlike other workers in harsh environments, they face synergistic, occupation-specific stressors: continuous rotating shifts (disrupting circadian homeostasis), simultaneous exposure to multiple hazards (noise, dust, toxic chemicals), remote isolation (limiting social support and mental health access), and high-stakes operations (inducing chronic psychological stress) (6–8)). These combined factors heighten vulnerability to occupational burnout, anxiety, and sleep disturbances. Recent petroleum industry studies confirm high prevalence of poor sleep and psychological distress in this group (9, 10), yet research on their interconnected relationships remains limited (11, 12).

Existing studies indicate that occupational burnout is closely associated with anxiety and poor sleep quality, yet the mechanisms underlying these relationships are not fully understood. Occupational burnout-related emotional exhaustion and depersonalization can heighten anxiety and activate physiological stress pathways, including the hypothalamic–pituitary–adrenal axis, which disrupts circadian regulation and sleep initiation (13, 14). Moreover, cross-sectional studies in industrial workers suggest that anxiety may serve as a psychological bridge linking occupational stress and poor sleep (15, 16). Nevertheless, most prior evidence derives from healthcare professionals or teachers, whereas data from industrial workers—particularly oilfield personnel—remain scarce (17).

In China, oilfield workers constitute a large and essential labor force engaged in continuous, high-intensity operations, yet occupational mental health surveillance in this group remains insufficient. Understanding the interconnections among occupational burnout, anxiety, and sleep quality is therefore critical for improving workforce sustainability and occupational health management (18, 19).

Accordingly, the present study aimed to (1) examine the associations among occupational burnout, anxiety, and sleep quality in oilfield workers; (2) explore whether anxiety mediates the association between occupational burnout and sleep quality; and (3) assess potential sex differences in these associations. This study provides new empirical evidence to inform targeted interventions that address both psychological and sleep-related health risks in high-demand industrial environments.

## 2 Materials and methods

### 2.1 Study setting and participants

This cross-sectional study was conducted between September and December 2022 among employees of an oilfield company in Shaanxi Province, China. A stratified random sampling approach was employed to ensure representative coverage across departments and job types. Specifically, one oilfield plant was randomly selected as the study site. Within this plant, several operation zones were randomly chosen to capture variations in work environments. Stratified random sampling was then conducted within each selected zone according to the male-to-female worker ratio to enhance representativeness.

A total of 1,745 workers were initially invited to complete the self-administered electronic questionnaire. After excluding non-respondents and questionnaires with missing or invalid responses, 1,617 valid samples were retained for analysis, yielding a response rate of 92.7%. Participants with missing data on key variables—exposure, mediator, or outcome indicators—were excluded from the final analytic dataset. As the proportion of missing data was less than 5%, no data imputation was applied.

Inclusion criteria were: (1) formal employment status, (2) continuous service of at least one year, and (3) provision of written informed consent. Individuals with a history of serious mental or organic illness or incomplete questionnaires were excluded.

### 2.2 Sample size estimation

To ensure adequate statistical power, the required sample size was calculated using a standard formula (1) for cross-sectional studies.


$$\begin{array}{l} N\;=\;\frac{{\left(Z_{1-\alpha }\right)}^{2}\;\times \;pq}{d^{2}} \end{array}$$ (1)

According to the formula: where p represents prevalence rate (22.57%) based on prior research (20), *q* equals 1 – *p*, α = 0.05, *Z*_1−α_ = 1.96, and d denotes permissible error (set at 0.10^*^p), the minimum required sample size N was calculated as 1,318. Accounting for sampling errors, the initially calculated sample size for simple random sampling was increased by 10%, resulting in a required sample size of 1,450. This study ultimately selected 1,617 oilfield workers as research subjects, which satisfies the sample size requirements.

### 2.3 Measurement of sleep quality, anxiety, and occupational burnout

Data were collected through a self-administered electronic questionnaire. Sleep quality was assessed using the Chinese version of the Pittsburgh Sleep Quality Index (PSQI) (21), with a global score >7 defined as poor sleep quality. Anxiety symptoms were measured via the SCL-90 anxiety subscale (22), with a factor score ≥2 indicating clinically relevant anxiety. Occupational burnout, the primary exposure variable, was assessed using the Chinese version of the Maslach Burnout Inventory–General Survey (MBI-GS) (23). According to the standard, add up the scores of each question and divide by 15 to obtain the average score, then multiply by 20 for judgment, for descriptive analysis, participants were categorized into “Occupation burnout” (MBI-GS ≥ 50) and “No occupational burnout” (MBI-GS <50) groups, based on prior Chinese occupational studies (24, 25).

All three instruments have been previously validated in Chinese occupational populations, including oilfield workers, and demonstrated good psychometric properties in the present sample. Specifically, the Cronbach's α coefficients were 0.935 for the MBI-GS, 0.882 for the PSQI, and 0.957 for the SCL-90 anxiety subscale, indicating excellent internal consistency. The Kaiser–Meyer–Olkin (KMO) values were 0.937, 0.902, and 0.953, respectively, showing good sampling adequacy for factor analysis. The cumulative variance contribution rates were 79.220%, 72.758%, and 73.061%, respectively, confirming satisfactory structural validity. These results suggest that the Chinese versions of the three scales were reliable and valid for measuring occupational burnout, sleep quality, and anxiety in this occupational population (Supplementary Table S1).

### 2.4 Covariates

Potential confounding factors included demographic variables (age, sex, education, marriage, ethnicity, religious belief, and income), lifestyle factors (smoking status, drinking status, and tea consumption), and occupational factors (occupation, night shift, chemical exposure, noise, and dust exposure). These variables were selected based on existing literature and the characteristics of oilfield work, aiming to capture three key domains (26–32): (1) occupational exposure factors (night shift work and exposure to chemicals, noise, and dust), which represent core stressors with well-documented associations with sleep and mental health; (2) lifestyle factors (body mass index, smoking, alcohol consumption, and tea drinking) that may confound mental and sleep outcomes through behavioral pathways; (3) sociodemographic factors (sex, age, education, marital status, and income) to adjust for background heterogeneity.

### 2.5 Statistical analysis

All analyses were performed using R software (version 4.4.3). Descriptive statistics were used to summarize baseline characteristics. Pearson correlation coefficients were calculated to examine the relationships between sleep quality, anxiety, and occupational burnout. To ensure model stability, multicollinearity among covariates was assessed using variance inflation factors (VIFs). All VIF values ranged from 1.05 to 1.59, which are well below the conventional threshold of 5, indicating no serious multicollinearity (Supplementary Table S2). Missing data for the exposure, outcome, or covariates (<5% overall) were handled using complete case analysis. Multiple logistic regression models were built to estimate the associations between occupational burnout and sleep quality, adjusting for potential confounders in a stepwise manner. Occupation burnout was modeled as a continuous variable using restricted cubic splines with three knots placed at the 10th, 50th, and 90th percentiles of the MBI-GS distribution, in accordance with established methodological guidelines (33, 34). This flexible approach enabled the detection of non-linear associations.

The full logistic regression model equation is provided below:


$$\begin{array}{l} logit\left(P_{i}\right)=\;\beta_{0}+\;f\left(MBI-GS_{i}\right)+\;\sum \beta_{j}X_{ij} \end{array}$$ (2)

Where *f* (MBI-GS*i*) represents the spline-transformed occupational burnout variable, and *Xij* denotes covariates.

To further explore potential effect modification, sex-stratified analyses and formal tests for interaction were conducted. Specifically, an interaction term between occupational burnout and sex was introduced into the fully adjusted logistic regression model to examine whether the association between occupational burnout and sleep quality differed by sex. Additionally, three quantitative indices were used to characterize the interaction effect: the relative excess risk due to interaction (RERI), attributable proportion due to interaction (AP), and synergy index (S).

The mediating effect of anxiety was examined using bootstrapped mediation analysis with 1,000 resamples. A two-sided *P*-value <0.05 was considered statistically significant.

## 3 Result

### 3.1 Participant characteristics

Among the 1,617 oilfield workers surveyed, 853 (52.75%) experienced occupational burnout (Table 1). The study population included 1,092 males (67.53%) and 525 females (32.47%), with a mean age of 40.67 ± 8.66 years. The average BMI was 24.09 ± 3.65 kg/m^2^, with 48.30% classified as overweight or obese. Occupational burnout rates differed significantly by age, marital status, occupation, and income (*P* < 0.05), but showed no significant variation across sex, BMI, education, ethnicity, religious beliefs, shift work, exposure to chemical, dust or noise hazards, smoking, alcohol, or tea consumption (*P* > 0.05). Among employees with occupational burnout, the prevalence of sleep quality (72.91%) and anxiety (13.25%) was markedly higher than that in the non-occupational burnout group (64.00% and 1.96%, respectively).

**Table 1:** Occupational burnout among employees with different basic characteristics.

**Characteristic**	**Non occupational burnout (*n* = 764)**	**Occupational burnout (*n* = 853)**	***P***
**Age, years**			<0.001
21–32	141 (18.45%)	263 (30.83%)	
33–39	175 (22.90%)	230 (26.96%)	
40–46	222 (29.05%)	182 (21.33%)	
47–59	226 (29.58%)	178 (20.86%)	
**Sex**			0.674
Male	512 (67.01%)	580 (67.99%)	
Female	252 (32.98%)	273 (32.00%)	
**Education**			0.705
Junior high school and below	75 (9.816%)	83 (9.73%)	
Normal high school	179 (23.42%)	193 (22.62%)	
Junior college	231 (30.23%)	242 (28.37%)	
Bachelor's degree or above	279 (36.51%)	335 (39.27%)	
**Marriage**			0.024
Unmarried	86 (11.25%)	136 (15.94%)	
Married	627 (82.06%)	664 (77.84%)	
Divorced/widowed	51(6.68%)	53 (6.21%)	
**Ethnicity**			0.911
Han	750 (98.16%)	838 (98.24%)	
Other	14 (1.832%)	15 (1.758%)	
**Religious belief**			0.345
No	733 (95.94%)	810 (94.95%)	
Yes	31 (4.06%)	43 (5.04%)	
**Income, yuan**			0.020
<100.000	162 (21.20%)	223 (26.14%)	
≥100.000	602 (78.79%)	630 (73.85%)	
**BMI**			0.135
Underweight	39 (5.10%)	25 (2.93%)	
Normal	364 (47.64%)	405 (47.47%)	
Overweight	267 (34.94%)	320 (37.51%)	
Obesity	94 (12.30%)	103 (12.07%)	
**Smoking status**			0.462
No	416 (54.45%)	480 (56.27%)	
Yes	348 (45.54%)	373 (43.72%)	
**Drinking status**			0.298
No	571 (74.73%)	618 (72.45%)	
Yes	193 (25.26%)	235 (27.54%)	
**Tea consumption**			0.937
No	503 (65.83%)	560 (65.65%)	
Yes	261 (34.16%)	293 (34.34%)	
**Occupation**			0.012
Oil recovery	347 (45.41%)	420 (49.23%)	
Station control	120 (15.70%)	160 (18.75%)	
Other	297 (38.87%)	273 (32.00%)	
**Night shift**			0.652
No	442 (57.85%)	484 (56.74%)	
Yes	322 (42.14%)	369 (43.25%)	
**Chemical exposure**			0.137
No	239(31.28%)	238(27.9%)	
Yes	525(68.71%)	615(72.09%)	
**Noise exposure**			0.134
No	367 (48.03%)	378 (44.31%)	
Yes	397 (51.96%)	475 (55.68%)	
**Dust exposure**			0.583
No	648(84.81%)	715(83.82%)	
Yes	116(15.18%)	138(16.17%)	
**Sleep quality**			<0.001
No	275 (35.99%)	231 (27.08%)	
Yes	489 (64.00%)	622 (72.91%)	
**Anxiety**			<0.001
No	749 (98.04%)	740 (86.75%)	
Yes	15 (1.96%)	113 (13.25%)	

### 3.2 Correlation between occupational burnout, sleep quality, and anxiety

**Figure 1:**
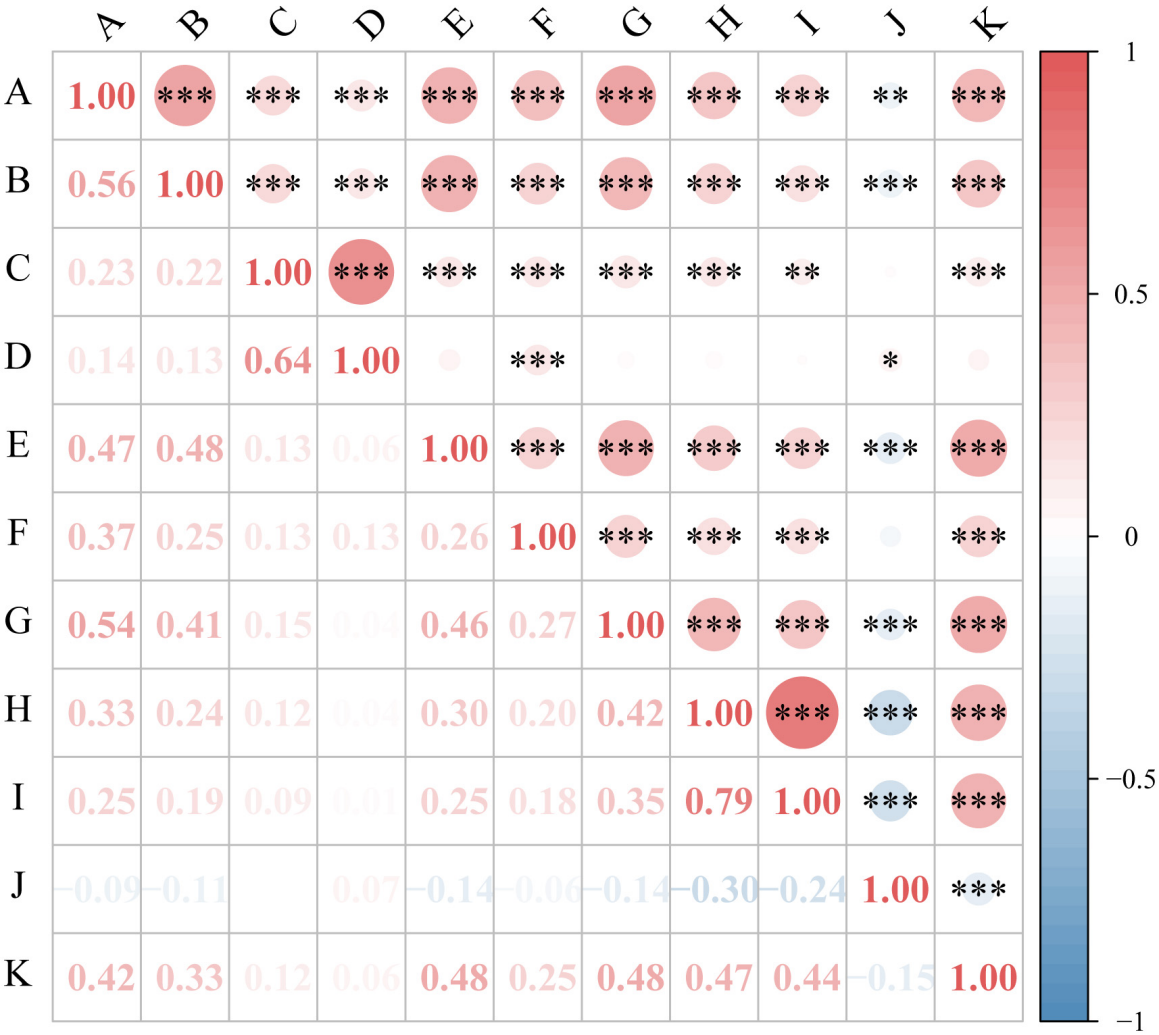
Correlations between occupational burnout, sleep quality, and anxiety. The figure presents a correlation matrix with values representing Pearson correlation coefficients. Red shading indicates positive correlations, blue indicates negative correlations, and color intensity reflects the strength of the correlation. A = sleep quality; B = sleep latency; C = sleep duration; D = sleep efficiency; E = sleep disturbances; F = use of hypnotic medication; G = daytime dysfunction; H = emotional exhaustion; I = depersonalization; J = reduced personal accomplishment; K = anxiety. *P < 0.05; **P < 0.01; ***P < 0.001.

To test the research hypothesis, Pearson correlation analysis was performed to examine the relationships among occupational burnout, sleep quality, and anxiety in oilfield workers. The results, presented in Figure 1, revealed significant positive correlations (*P* < 0.05) between six dimensions of sleep quality (sleep quality, sleep latency, sleep duration, sleep disturbances, use of sleep medication, and daytime dysfunction) and all three dimensions of occupational burnout (emotional exhaustion, depersonalization, and reduced personal accomplishment). However, sleep efficiency showed a significant negative correlation with reduced personal accomplishment (*P* < 0.05). Additionally, all dimensions of occupational burnout were positively correlated with anxiety (*P* < 0.05), whereas all seven dimensions of sleep quality were negatively correlated with anxiety (*P* < 0.05). These findings suggest potential interconnections among occupational burnout, anxiety, and sleep quality in this population.

### 3.3 The association between occupational burnout and sleep quality

Occupational burnout were significantly associated with sleep quality across all models (Table 2). In the total population, workers with occupational burnout had higher odds of reporting poor sleep quality in the crude analysis (Model 1: *OR* = 1.514, 95% CI: 1.226–1.870), and the association became slightly stronger after adjusting for demographic factors and BMI (Model 2: *OR* = 1.599, 95% CI: 1.284–1.993). The relationship remained robust after further controlling for occupational and lifestyle factors (Model 3: *OR* = 1.611, 95% CI: 1.291–2.010). Sex-stratified analyses yielded consistent findings, with females showing stronger associations (*OR* = 1.919, 95% CI: 1.244–2.959) compared with males (*OR* = 1.492, 95% CI: 1.144–1.946). These results suggest that females may be more vulnerable to the detrimental association of sleep disturbances on occupational burnout. To formally test sex differences, an interaction term between occupational burnout and sex was included in the logistic model. Three key indices further quantified their interaction: RERI stood at 0.609, indicating excess risk beyond the sum of individual effects of the two factors; AP was 0.256, meaning 25.600% of occupational burnout risk in co-exposed groups was attributable to their interaction; S reached 1.788, confirming a synergistic effect where their combined impact exceeded the sum of separate effects.

**Table 2:** Association between occupational burnout and sleep quality.

**Sleep quality**	**Occupational burnout**		
	**No**	**Yes**	
		**OR (95% CI)**	***P***
**Total population**			
Model 1	1.000 (Reference)	1.514 (1.226, 1.870)	<0.001
Model 2	1.000 (Reference)	1.599 (1.284, 1.993)	<0.001
Model 3	1.000 (Reference)	1.611 (1.291, 2.010)	<0.001
**Female**			
Model 1	1.000 (Reference)	1.836 (1.236, 2.725)	0.003
Model 2	1.000 (Reference)	1.864 (1.217, 2.855)	0.004
Model 3	1.000 (Reference)	1.919 (1.244, 2.959)	0.003
**Male**			
Model 1	1.000 (Reference)	1.412 (1.098, 1.816)	0.007
Model 2	1.000 (Reference)	1.494 (1.147, 1.946)	0.003
Model 3	1.000 (Reference)	1.492 (1.144, 1.946)	0.003

Model 1: Unadjusted; Model 2: Adjust for age, sex, education, marriage, ethnicity, religious belief, occupation, income, and BMI; Model 3: Further adjust for night shift, chemical exposure, noise exposure, dust exposure, smoking, drinking status, and tea consumption, with a test standard of *P* < 0.05. Sex stratification analysis does not adjust for sex.

**Figure 2:**
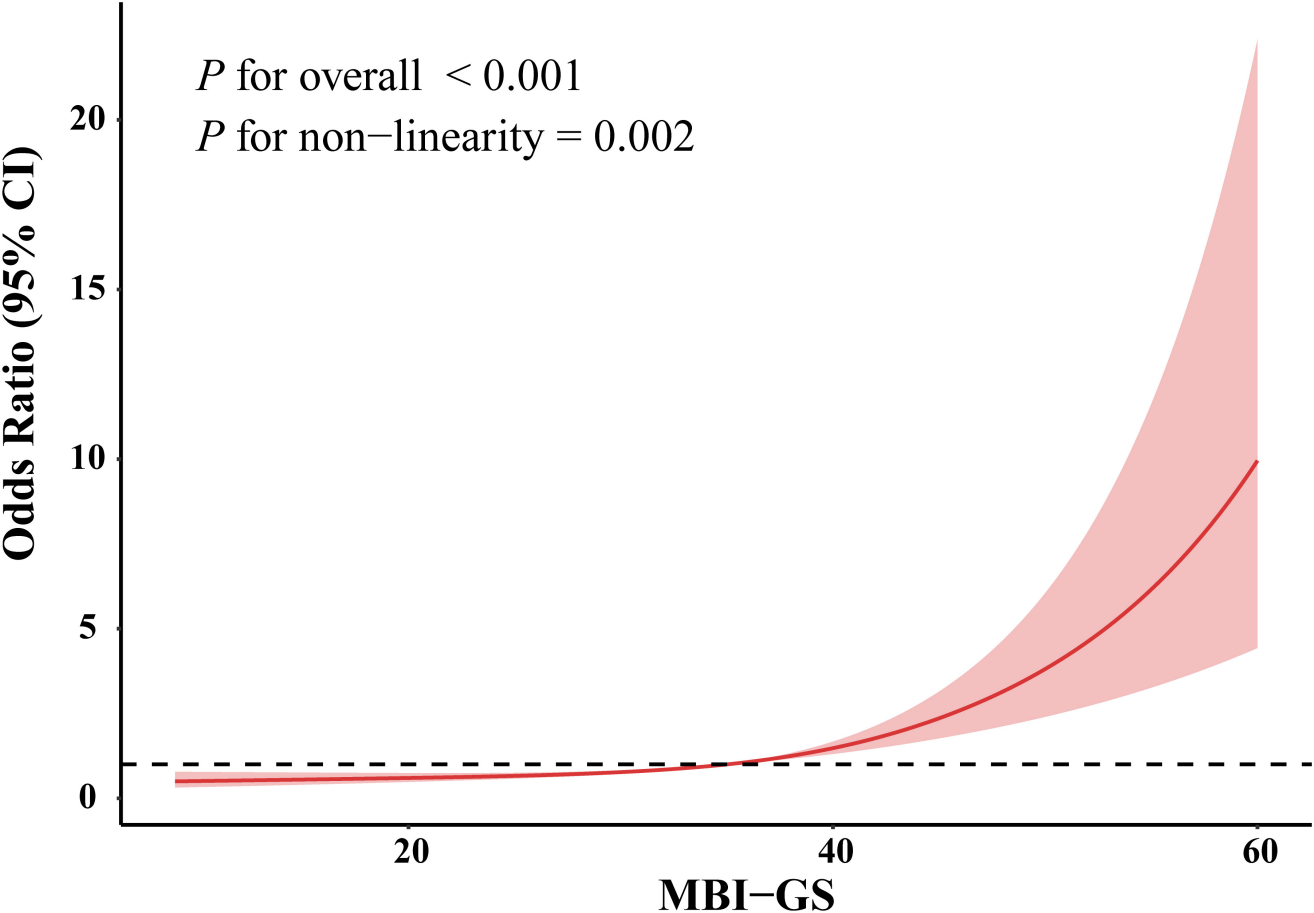
The relationship between occupational burnout and sleep quality. The red curve represents the odds ratio (95% CI) for occupational burnout associated with MBI-GS score, derived from restricted cubic spline regression. Shaded areas represent the 95% confidence interval.

Restricted cubic spline analysis further indicated a nonlinear association pattern between occupational burnout and the odds of poor sleep quality (*P* for overall <0.001; *P* for nonlinear = 0.002) (Figure 2). The odds of experiencing occupational burnout remained stable at lower MBI-GS values but increased sharply once MBI-GS scores exceeded about 40, suggesting a threshold association. This finding highlights the marked increase in occupational burnout odds associated with severe sleep disturbances, suggesting the clinical relevance of early occupational burnout intervention for mitigating the likelihood of sleep problem.

### 3.4 The association between occupational burnout and anxiety

As shown in Table 3, employees with occupational burnout were significantly more likely to experience anxiety. In the overall sample, the odds of anxiety were 7.625 times higher in unadjusted analyses (Model 1, 95% CI: 4.408–13.191, *P* < 0.001) and remained elevated after full adjustment (Model 3: *OR* = 7.832, 95% CI: 4.473–13.711, *P* < 0.001). When examined by sex, male employees exhibited a stronger association (Model 3: *OR* = 9.289, 95% CI: 4.676–18.452) compared with female employees (Model 3: *OR* = 5.104, 95% CI: 1.868–13.946). These findings highlight that occupational burnout is a substantial strongly with increased odds for anxiety, with a more pronounced association observed among men.

**Table 3:** Correlation analysis between occupational burnout and anxiety.

**Anxiety**	**Occupational burnout**		
	**No**	**Yes**	
		**OR (95% CI)**	***P***
**Total population**			
Model 1	1.000 (Reference)	7.625 (4.408, 13.191)	<0.001
Model 2	1.000 (Reference)	7.618 (4.363, 13.299)	<0.001
Model 3	1.000 (Reference)	7.832 (4.473, 13.711)	<0.001
**Female**			
Model 1	1.000 (Reference)	6.099 (2.328, 15.979)	<0.001
Model 2	1.000 (Reference)	4.922 (1.822, 13.294)	0.002
Model 3	1.000 (Reference)	5.104 (1.868, 13.946)	<0.001
**Male**			
Model 1	1.000 (Reference)	8.384 (4.300, 16.346)	<0.001
Model 2	1.000 (Reference)	8.876 (4.496, 17.524)	<0.001
Model 3	1.000 (Reference)	9.289 (4.676, 18.452)	<0.001

Model 1: Unadjusted; Model 2: Adjust for age, sex, education, marriage, ethnicity, religious belief, occupation, income, and BMI; Model 3: Further adjust for night shift, chemical exposure, noise exposure, dust exposure, smoking, drinking status, and tea consumption, with a test standard of *P* < 0.05. Sex stratification analysis does not adjust for sex.

### 3.5 The association between sleep quality and anxiety

Table 4 presents the association between anxiety and sleep quality. Across all models, anxiety was strongly linked to an increased odds of sleep disturbances. In the overall sample, employees with anxiety had 5.894 times higher odds of having sleep quality in the unadjusted model (Model 1, 95% CI: 3.063–11.341, *P* < 0.001), and this association remained significant after full adjustment (Model 3, *OR* = 6.077, 95% CI: 3.142–11.757, *P* < 0.001).

**Table 4:** Correlation analysis between sleep quality and anxiety.

**Anxiety**	**Sleep quality**		
	**No**	**Yes**	
		**OR (95% CI)**	***P***
**Total population**			
Model 1	1.000 (Reference)	5.894 (3.063, 11.341)	<0.001
Model 2	1.000 (Reference)	6.216 (3.216, 12.012)	<0.001
Model 3	1.000 (Reference)	6.077 (3.142, 11.757)	<0.001
**Female**			
Model 1	1.000 (Reference)	6.275 (1.485, 26.509)	<0.001
Model 2	1.000 (Reference)	5.944 (1.363, 25.93)	0.018
Model 3	1.000 (Reference)	5.679 (1.293, 24.938)	0.021
**Male**			
Model 1	1.000 (Reference)	6.012 (2.879, 12.553)	<0.001
Model 2	1.000 (Reference)	6.282 (2.980, 13.247)	<0.001
Model 3	1.000 (Reference)	6.207 (2.940, 13.105)	<0.001

Model 1: Unadjusted; Model 2: Adjust for age, sex, education, marriage, ethnicity, religious belief, occupation, income, and BMI; Model 3: Further adjust for night shift, chemical exposure, noise exposure, dust exposure, smoking, drinking status, and tea consumption, with a test standard of *P* < 0.05. Sex stratification analysis does not adjust for sex.

When stratified by sex, both male and female employees with anxiety showed elevated odds of poor sleep quality. In Model 3, the odds were 6.207 (95% CI: 2.940–13.105, *P* < 0.001) for males and 5.679 (95% CI: 1.293–24.938, *P* = 0.021) for females, indicating a slightly stronger association among male employees. These results consistently suggest that anxiety is an independently associated factor of sleep disturbances in the workplace population.

### 3.6 Mediation effect analysis

Bootstrap mediation analysis revealed an association structure consistent with a significant indirect relationship between occupational burnout and sleep quality via anxiety (Supplementary Table S3). The indirect component through anxiety accounted for 33.08% of the total association, while the direct component accounted for 66.92% (95% CI: 0.025–0.114 for direct component; 0.022–0.049 for indirect component), suggesting a partial mediation pattern.

## 4 Discussion

This study identified strong associations among occupational burnout, anxiety, and sleep quality among oilfield workers, extending prior research conducted in other occupational groups. Our findings revealed that workers with higher occupational burnout levels were more likely to report poor sleep quality, and that anxiety accounted for approximately one-third of this association. These results are consistent with evidence from healthcare and industrial workers showing that occupational burnout is a significant correlate of sleep impairment and psychological distress (35–37).

The mediation effect of anxiety reflects a theoretically grounded dual pathway. Psychologically, burnout's core symptom—emotional exhaustion—triggers persistent negative affect and helplessness, exacerbating anxiety and cognitive hyperarousal (e.g., rumination about work risks) that directly disrupts sleep onset (38). Physiologically, chronic burnout activates the hypothalamic–pituitary–adrenal (HPA) axis, leading to dysregulated cortisol secretion; anxiety further amplifies this HPA overactivation, blunting diurnal cortisol rhythms and impairing circadian sleep-wake cycles. This aligns with prior findings in construction workers and nurses (39). but extends to oilfield workers, where occupational hazards and shift work may strengthen this pathway. Clinically, this means interventions targeting sleep are incomplete—addressing anxiety can break the burnout-anxiety-sleep disturbance cycle (40, 41).

The magnitude of the associations observed in our study appears stronger than those reported among healthcare or administrative personnel, likely reflecting the compounded influence of physical workload, shift rotation, and environmental exposures inherent to oilfield operations . For example, workers in Indonesian petroleum industries exhibited elevated anxiety and sleep quality scores compared with non-industrial employees (42). These contextual stressors may amplify occupational burnout symptoms and their downstream effects on sleep quality.

Our sex-stratified analyses revealed that female workers exhibited stronger associations between burnout and poor sleep quality, consistent with prior evidence that women experience greater emotional exhaustion and stress sensitivity (31, 43). Possible explanations include hormonal fluctuations affecting sleep–wake regulation and the additional burden of family-related responsibilities. This finding highlights the importance of sex-sensitive occupational health interventions.

The high prevalence of occupational burnout and sleep problems among oilfield workers underscores the need for integrated workplace health promotion programs. Strategies should include optimizing shift schedules, providing psychological counseling and relaxation training, and improving the psychosocial work environment (44, 45). Regular screening for anxiety and occupational burnout symptoms could help identify at-risk employees early. Moreover, management-level interventions—such as ensuring adequate rest periods and reducing excessive overtime—may effectively alleviate fatigue and sleep quality (46).

However, this study has several limitations that should be acknowledged. First, as a cross-sectional survey, this design fundamentally restricts our ability to infer causality. Future longitudinal studies are therefore needed to examine the temporal order among these variables, and more longitudinal data collection as well as prospective cohort studies will be necessary to empirically verify potential causal relationships. Second, the interpretation of the bootstrapped mediation analysis is inherently constrained by the cross-sectional design. Specifically, the model relies on the theoretical assumption of a temporal sequence (with occupational burnout preceding anxiety, which in turn precedes sleep disturbances), an assumption that cannot be empirically confirmed in this study. This limitation renders the findings susceptible to reverse causation, as well as potential unmeasured confounding between the proposed mediator (anxiety) and the outcome (sleep quality). Therefore, the mediating components must be interpreted cautiously: they reflect an association structure rather than conclusive evidence of a causal pathway. Third, data were mainly collected through questionnaire surveys, with information on occupational burnout, anxiety levels, and sleep quality being self-reported; this may introduce recall bias or subjective bias. Fourth, the study population was drawn from a single oilfield in Shaanxi Province, which may limit the external validity and generalizability of the findings. Working conditions, shift patterns, and psychosocial environments can vary substantially across regions and industries, and these contextual differences may influence both occupational burnout and sleep outcomes. Therefore, caution should be exercised when extrapolating the present results to other oilfields or occupational groups. Future multi-center studies conducted across diverse geographic and organizational settings are recommended to validate and extend these findings. Finally, the main research subjects are specific occupational groups. When extending the research results to other occupational types or a wider population, further validation and supplementary research are required. In addition, some potentially important covariates—such as detailed work schedules, psychosocial work conditions, and physical comorbidities—were not captured in this survey, which may introduce residual confounding. Future longitudinal studies should incorporate these variables to further strengthen causal inference.

## 5 Conclusion

This study highlights a significant relationship between occupational burnout, anxiety, and sleep quality among oilfield workers. Our analysis revealed that occupational burnout was directly associated with sleep disturbances, and there was an additional indirect association through anxiety. This indirect pathway explained approximately 33.08% of the total association, aligning with a mediating role of anxiety. These findings underscore the importance of workplace interventions targeting both occupational burnout and mental health.

## Data Availability

The raw data supporting the conclusions of this article will be made available by the authors, without undue reservation.
