# Supplementary material for: Interconnected associations of occupational burnout, anxiety, and sleep quality in oilfield workers
Source: Front Public Health. 2026 Jan 12;13:1723075. doi: 10.3389/fpubh.2025.1723075 (PMC12850518; doi:10.3389/fpubh.2025.1723075)
Supplement: Supplementary file 1 [file Data_Sheet_1.pdf]

Table S1 Cronbach's α, KMO, and Cumulative Variance Contribution Rate of Each Scale.

Instrument	Cronbach's α	KMO	Cumulative variance contribution (%)
**MBI-GS (Burnout)**	0.935	0.937	79.220
**PSQI (Sleep Quality)**	0.882	0.902	72.758
**SCL-90 (Anxiety Subscale)**	0.957	0.953	73.061

Table S2 Testing for multicollinearity among the included covariates.

Variable	Variance Inflation Factors
Age	1.571404
Sex	1.190005
Education	1.358627
Marriage	1.280280
Ethnicity	1.112597
Religious belief	1.127904
Income	1.047128
BMI	1.146963
Smoking status	1.324209
Drinking status	1.321357
Tea consumption	1.191009
Occupation	1.052507
Night shift	1.123648
Chemical Exposure	1.412476
Noise Exposure	1.585937
Dust Exposure	1.168045

Table S3 The mediating role of anxiety between occupational burnout and sleep quality.

Model effect	Effect value	Bootstrap 95%CI		Proportion of Effect
		Lower limit	Upper limit	
**Total effect**	0.105	0.060	0.149	100.00%
**Direct effect**	0.070	0.025	0.114	66.92%
**Indirect effects**	0.035	0.022	0.049	33.08%

CI, Confidence Interval.
